# Determination of the intracellular concentration of the export chaperone SecB in *Escherichia coli*

**DOI:** 10.1371/journal.pone.0183231

**Published:** 2017-08-29

**Authors:** Bahar T. Findik, Linda L. Randall

**Affiliations:** Department of Biochemistry, University of Missouri, Columbia, Missouri, United States of America; Centre National de la Recherche Scientifique, Aix-Marseille Université, FRANCE

## Abstract

SecB, a small tetrameric chaperone in *Escherichia coli*, plays a crucial role during protein export via the general secretory pathway by binding precursor polypeptides in a nonnative conformation and passing them to SecA, the ATPase of the translocon. The dissociation constants for the interactions are known; however to relate studies *in vitro* to export in a living cell requires knowledge of the concentrations of the proteins in the cell. Presently in the literature there is no report of a rigorous determination of the intracellular concentration of SecB. The values available vary over 60 fold and the details of the techniques used are not given. Here we use quantitative immunoblotting to determine the level of SecB expressed from the chromosome in *E*.*coli* grown in two commonly used media. In rich medium SecB was present at 1.6 ± 0.2 μM and in minimal medium at 2.5 ± 0.6 μM. These values allow studies of SecB carried out *in vitro* to be applied to the situation in the cell as SecB interacts with its binding partners to move precursor polypeptides through the export pathway.

## Introduction

The general secretory (Sec) system of *E*. *coli* functions to localize proteins to the inner membrane, the periplasm and the outer membrane [1]. Export via this system requires that precursor polypeptides be devoid of stable tertiary structure [2]. In the cytoplasm a small tetrameric chaperone, SecB (molar mass of tetramer 68.6 kDa), acts early in the export process by binding precursor polypeptides before they acquire a stable tertiary structure and maintaining them in an unfolded state for translocation across the cytoplasmic membrane [3–5]. Eight proteins were originally identified that utilize SecB for export [3, 6–10]. An additional ten proteins were discovered to use SecB through a comparative proteomic approach [11]. SecB binds with low specificity to the mature portion of a wide variety of ligands. The low specificity binding is of high affinity [12] characterized by high rates of association and dissociation [13, 14]. SecB directs its bound ligand to the transmembrane channel formed by SecYEG, a heterotrimeric protein assembly via interaction with SecA. SecA exists both free in the cytoplasm and membrane bound [15] in complex with the translocon SecYEG. Multiple dynamic interactions are involved as a precursor moves from SecB to SecA and on through the translocon.

The dissociation constants (K_d_s) of most of the interactions involving SecB have been determined [16–23]. The extent of binding between two proteins is a function of the K_d_ of the complex relative to the concentration of the proteins that associate to form that complex; therefore, to apply what we learn from studies *in vitro* to processes *in vivo* we must know the concentrations of the proteins within the *E*. *coli* cell. The numbers of copies of each protein of the Sec system within an individual cell of *E*. *coli* has been determined and, with the exception of SecB, are given in Table 1. We calculated the concentration of SecA, one of the binding partners of SecB, as 5 μM using the average of the published values for the number of copies per cell and our finding here that the volume of cytoplasmic water in one cell is 1.3 femtoliter. Since as much as 50% of SecA in the cell is associated with the cytoplasmic membrane [15] the soluble SecA would be approximately 2.5 μM. There are four reports in the literature of the cellular concentration of SecB. Each estimate has significant limitations. The first, published by Hardy and Randall [12], was a rough estimate based on the yield of a published purification of SecB [24]. The details of the purification were not given, thus many assumptions were made in arriving at an estimate of 4 μM SecB tetramer. Two other values, 20 μM [25] and 13 μM [26], were cited as unpublished. In both cases there is no mention of the techniques used to determine the concentrations. Seoh and Tai [27] used immunoblotting and showed that the level of SecB in a cell was dependent on the carbon source during growth. When cells were grown in the presence of glucose the number of copies of SecB per cell was approximately 1000. This is equivalent to 0.2 μM tetramer if the volume of a cell is taken as 1.3 femtoliter. The level of SecB was two-fold higher for growth on glycerol [27]. There are two proteomic studies in the literature that report the abundance of SecB and several other proteins of the export apparatus (see Table 1 for proteins other than SecB). One study is aimed at the establishment of condition-dependent proteome profiles for systems studies [28]. They examined three strains of *E*. *coli* under 22 experimental conditions. Using the number of copies of SecB per cell reported in that work and the cellular volumes determined here (see Table 2), we calculate the intracellular concentration of SecB tetramer to be 5.5 μM (6,100 copies tetramer) in rich medium and 2.5 μM (3,200 copies tetramer) in minimal medium. This is consistent with the two-fold difference observed by Seoh and Tai that results from catabolite repression [29]. The other study [30] used MC4100, the strain used in this study. The cells were grown both in rich and in minimal media, but the data were combined for analysis. This work reported the number of copies of SecB per cell as 3190 protomers, equivalent to 0.7 μM tetramer, calculated using 1.2 μl as the volume of a cell, i.e., the average of the cytoplasmic volumes determined here for growth in rich and in minimal medium. Because of the variation in strains used, in growth condition and in the published estimates, we have re-examined the question of the concentration of SecB *in vivo*.

**Table 1:** Proteins of the Sec system.

Protein	SecA	SecY	SecE	SecG	SecD	SecF
**Copies/cell**	2500–5000^a^ 500^e^ 1800^f^	200–400^a^ 1300^e^	300–600^a^ 250–500^b^	650–1300^c^	7–30^d^ 600^e^	30–60^a^ 7–30^d^ 200^e^

^a^ [31] and ^c^ [32] Determined from the intensity of Coomassie Brilliant Blue stain on SDS-polyacrylamide gels.

^b^ [33] Determined from measurement of alkaline phosphatase activity of a *secE-phoA* fusion.

^d^ [34] Determined from measurement of alkaline phosphatase activity of a *secD-phoA* fusion.

^e^ [30] Determined by mass spectrometry.

^f^ [28] Determined by mass spectrometry.

**Table 2:** Parameters used for calculations of molar concentration of SecB.

Growth Medium	Osmolality^a^ (Osm)	Cell/ml^b^ at 1 OD	SecB /cell^c^ (x 10^−16^ g)	V_cell_^d^ (x 10^−15^ L)	Vcyto^e^ (x 10^−15^ L)	SecB Molarity ^c^ (μM tetramer)
M9B1-Glycerol	0.29	1.5 ± 0.4 x 10^9^	2.2 ± 0.6	1.5	1.3	2.5 ± 0.6
LB	0.42	1.1 ± 0.2 x 10^9^	1.2 ± 0.1	1.4	1.1	1.6 ± 0.2

^a^ Determined by dew-point temperature depression as described in Materials and methods.

^b^ Determined using a Petroff-Hausser counting chamber as described in Materials and methods.

^c^ The average from 3 independent cultures, see text for an example of calculation.

^d^ Taken from data in Table 1 of Cayley et al. [39].

^e^ Calculated using the ratio of V_cyto_ to V_cell_ in Fig 2 of Cayley et al. [40].

## Materials and methods

### Bacterial strains and plasmids

A large number of studies of SecB *in vivo* have been carried out in the *E*. *coli* K12 strain MC4100 (F^−^*araD139 Δ(argF-lac)U169 rpsL150* (Str^R^)*relA1 flbB5301 deoC1 ptsF25 rbsR*). Therefore MC4100 was used in this study. MC4100 carrying a *secB* null mutation, Δ*secB*::*Cm*^*R*^ [35] that does not have polarity on *gpsA* [36] was a gift from Pierre Genevaux. Wild-type SecB was purified from the *E*. *coli* B strain BL21(DE3) (F^−^*ompT gal dcm lon hsdS*_*B*_(*r*_*B*_^−^*m*_*B*_^−^) λ(DE3 [*lacI lacUV5*-*T7p07 ind1 sam7 nin5*]) [*malB*^+^]_K-12_(λ^S^)), harboring plasmid pJW25, which carries wild-type *secB* under T7 RNA polymerase and *bla* for ampicillin resistance.

### SecB purification

SecB used as a standard for immunoblots was purified as previously described [37]. The cells were disrupted using a French press at 8,000 psi. The cellular lysate was centrifuged at 362,000 × g in a 60Ti rotor (Beckman) for 3h at 4°C. SecB was purified from the high-speed supernatant using a QAE anion exchange column (TosoHaas). The concentration of SecB was determined spectrophotometrically at 280 nm using 47,600 M^-1^ cm^-1^ as the extinction coefficient for the SecB tetramer.

### Cell growth

To determine the intracellular concentration of SecB, three independent cultures of MC4100 and of the *secB* null strain, MC4100Δ*secB*::*Cm*^*R*^, were grown both in M9 minimal medium supplemented with 0.4% (wt wt^-1^) glycerol and 4 μg ml^-1^ vitamin B1 and in the rich medium LB (Luria-Bertani, also called Lysogeny Broth) [38]. Optical densities were measured at 560 nm. Cells were inoculated to an optical density between 0.08 and 0.1 and grown at 35°C with shaking. The doubling times were 30 minutes in LB medium and 75 minutes in minimal medium. When the culture reached an optical density of between 1 and 2, growth was stopped by swirling on ice. The cultures were frozen by dripping into liquid nitrogen and stored at -70°C until used for analyses.

### Osmolality of growth media

The osmolality of each growth medium was measured by the method of dew-point temperature depression using a VAPRO osmometer 5520 (Wescor Inc., Logan, UT).

### Determination of cell number

The number of cells per ml (cells ml^-1^) at an optical density of 1.0 at 560 nm was determined for each culture using a Petroff-Hausser counting chamber (Hausser Scientific Company, Horsham, PA). After dilution of the culture to an optical density of 8 × 10^−3^ using the growth medium, the culture was applied to the counting chamber. The slide was viewed at a magnification of 400. For each determination the cells in 64 squares, giving a combined volume of 8 × 10^−2^ μl, were counted. The number of cells at an optical density of 8 × 10^−3^ was converted to cells ml^-1^ at an optical density of 1.0 for use in the calculations described in the text. The number of cells ml^-1^ given in Table 2 are the average of five determinations from three independent cultures for minimal medium and the average of eleven determinations from seven independent cultures for rich medium.

### TCA precipitation of SecB at high and low concentration

^14^C-SecB was precipitated at two different concentrations: 80 μg ml^-1^ (HIGH) and 0.34 μg ml^-1^ (LOW) by addition of trichloroacetic acid (TCA) to a final concentration of 11% (wt wt^-1^). The precipitates were collected by centrifugation (16,000 × g, 15 min at 10°C, Eppendorf centrifuge), washed with acetone and suspended in sample buffer. ^14^C-SecB that had not been precipitated with TCA was loaded on 14% (wt vol^-1^) polyacrylamide gels (0.54 mm thickness) for SDS-polyacrylamide gel electrophoresis at different concentrations to prepare a standard. The TCA precipitated samples were loaded to same amount assuming 100% recovery. The quantities of ^14^C-SecB on the gels were measured using a Fujifilm FLA 3000 phosphorimager in the linear range of its response.

### Sample preparation for Western blots

Portions of the frozen cultures were thawed and precipitated by addition ofTCA (11% wt wt^-1^). The precipitate was collected by centrifugation (16,000 × g, 15 min at 10°C, Eppendorf centrifuge), washed with acetone and suspendedin sample buffer to a final optical density at 560 nm of between 15 and 20 and subjected to electrophoresis on SDS-PAGE, 14% (wt vol^-1^) polyacrylamide gels (0.5 mm thickness).

To generate standard curves purified SecB was mixed with a culture of the *secB* null strain in an amount equal to that in the samples to be quantified. TCA precipitation was carried out immediately to avoid proteolysis and the samples were processed for gel electrophoresis as described above.

### Quantitative immunoblotting

After electrophoresis, proteins were transferred from the gel to a nitrocellulose membrane (pore size: 0.45 μm) for 2 h at 12 V. The membrane was immersed in 2% (wt vol^-1^) powdered milk in 20 mM Tris-HCl, pH 7.0, 0.5 M NaCl (PM-TBS) and placed on a shaker at 35°C for 15 min. After pouring off the milk, rabbit antiserum specific for SecB was added at a dilution of 1:1000 in PM-TBS and the membrane was incubated on a shaker at 35°C for 2 h. The antiserum was poured off and the membrane was washed with TWEEN, 0.05% (vol vol^-1^) in PM-TBS for 15 min. The membrane was then immersed in a 1:2000 dilution of the secondary antibody, goat antiserum raised to rabbit immunoglobulin G, conjugated with horseradish peroxidase. The washing step was repeated and the blot was developed with a solution of 4-chloro-1-napthol. Fifteen milligrams of 4-chloro-1-napthol in 5 ml of methanol were mixed with 25 ml TBS, 15 μl of 30% H_2_O_2_ were added immediately before use, and the blot was developed for 20 min. The dried membranes were photographed using a KODAK EDAS 290 digital camera system. The intensities of the bands were quantified using TotalLab. The standard curve of pure SecB used for quantitation was present on the same blot as the samples of culture under analysis.

## Results and discussion

**Fig 1:**
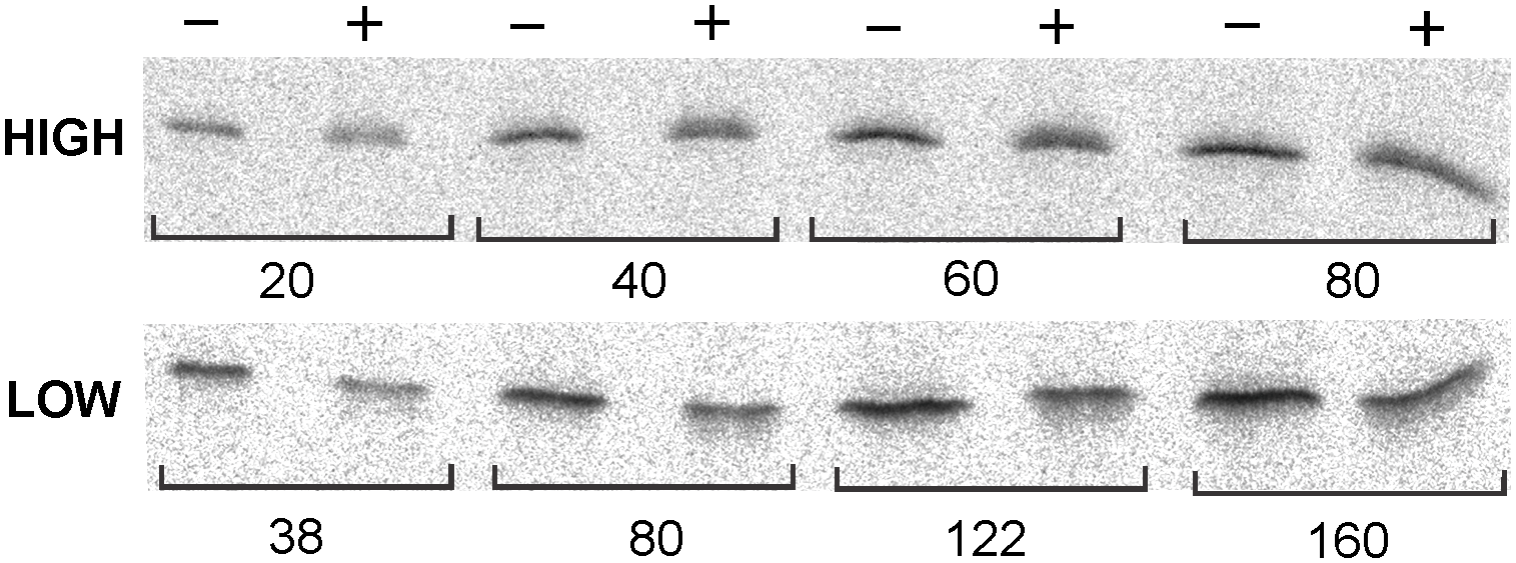
Recovery after TCA precipitation of SecB at high and low concentration. ^14^C-SecB was precipitated by addition of TCA (11% wt wt^-1^) at two different concentrations: 80 μg ml^-1^ **(HIGH)** and 0.34 μg ml^-1^ **(LOW)**. SecB was loaded after suspension in sample buffer either without (**−**) or after (**+**) TCA precipitation. The quantity of SecB loaded is given under the lanes in ng.

**Fig 2:**
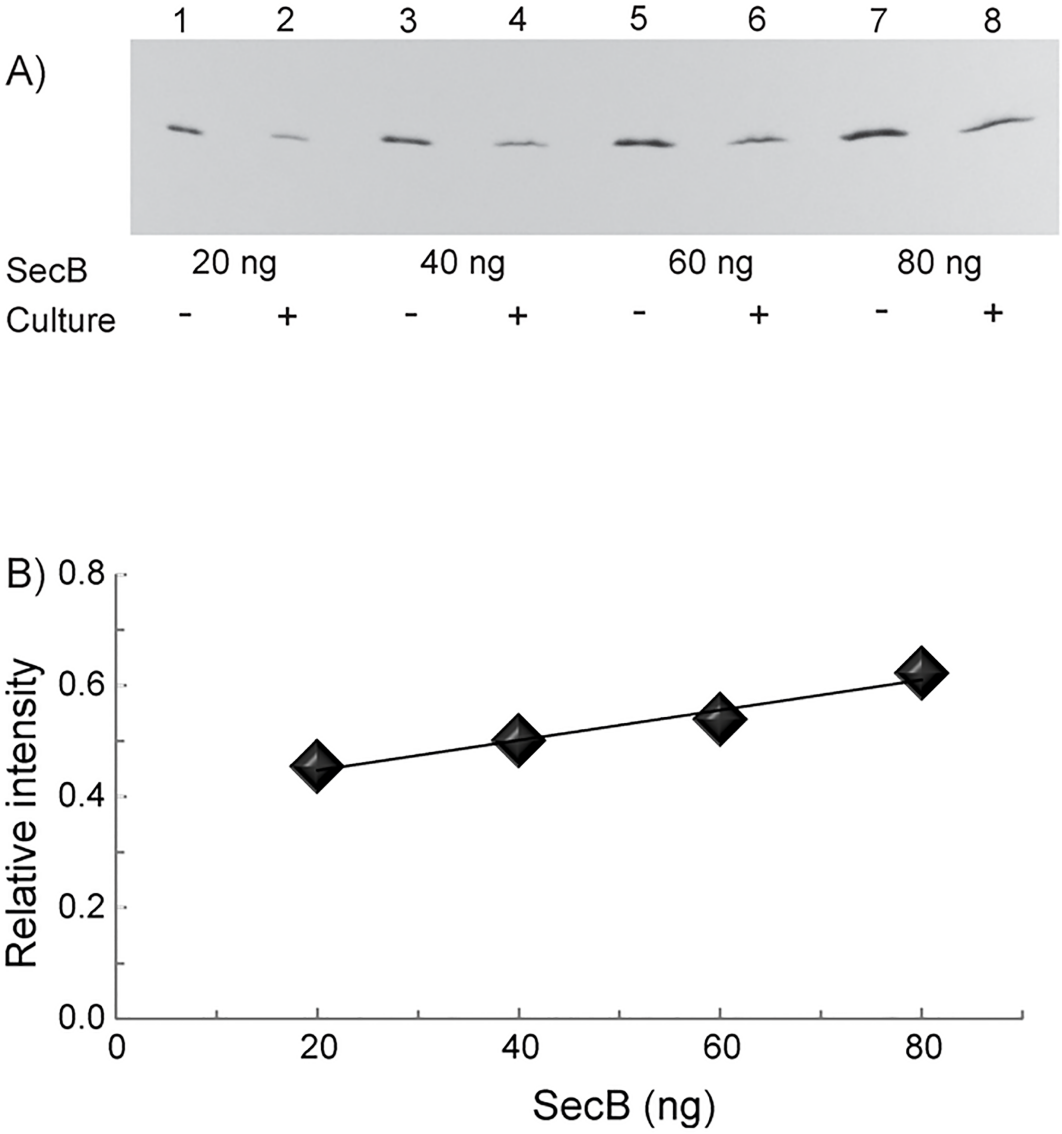
Effect of total cell culture on immunoblot intensity. **(A)** Lanes 1, 3, 5 and 7 were loaded with pure SecB at 20, 40, 60 and 80 ng after TCA precipitation at the concentration of 0.34 μg ml^-1^. Lanes 2, 4, 6 and 8 were loaded with the same quantity of pure SecB mixed with a culture of a *secB* null strain at optical density 15 before TCA precipitation. (**B)** Comparison of the intensity of the bands of SecB precipitated in the presence of cellular contents with those representing the same quantity of SecB precipitated without cellular contents, shown in **(A)**.

We determined the concentration of SecB expressed at the chromosomal level in a wild-type strain of *E*. *coli* K12 by quantitative immunoblotting. In order to minimize loss of protein, we avoided manipulations such as cell lysis and isolation of the soluble fraction. We added trichloroacetic acid (TCA) directly to a quantity of cell culture that contained sufficient SecB so that reactions on immunoblots were in the linear range of sensitivity. The recovery of pure SecB after TCA precipitation is concentration dependent (Fig 1). Precipitation at the high concentration (80 μg ml^-1^) gave excellent recovery, approximately 96% (Fig 1, HIGH). However, when SecB was precipitated by TCA at the low concentration (0.34 μg ml^-1^), which is in the range estimated for SecB in a lysate, the recovery was approximately 53% (Fig 1, LOW). After TCA precipitation, the relative intensities on the immunoblots varied with the ratio of the amount of SecB to the amount of cell culture present (Fig 2A). We cannot distinguish changes in efficiency of TCA precipitation of SecB in lysates from the effects of the presence of lysate on the reactions involved in developing the immunoblots; nonetheless, it is clear that the sensitivity of the immunoblot is affected by the cellular contents in the sample (Fig 2A and 2B). In the presence of a cell culture of a *secB* null strain at an optical density of 15, the reaction with 20 ng of pure SecB was 45% of that seen with pure SecB alone and at 80 ng of SecB it was 62%. Therefore, to generate standard curves for each of the samples a known quantity of pure SecB was precipitated by TCA after it was mixed with an amount of the *secB* null culture equal to the amount of the culture in the sample to be quantified.

**Fig 3:**
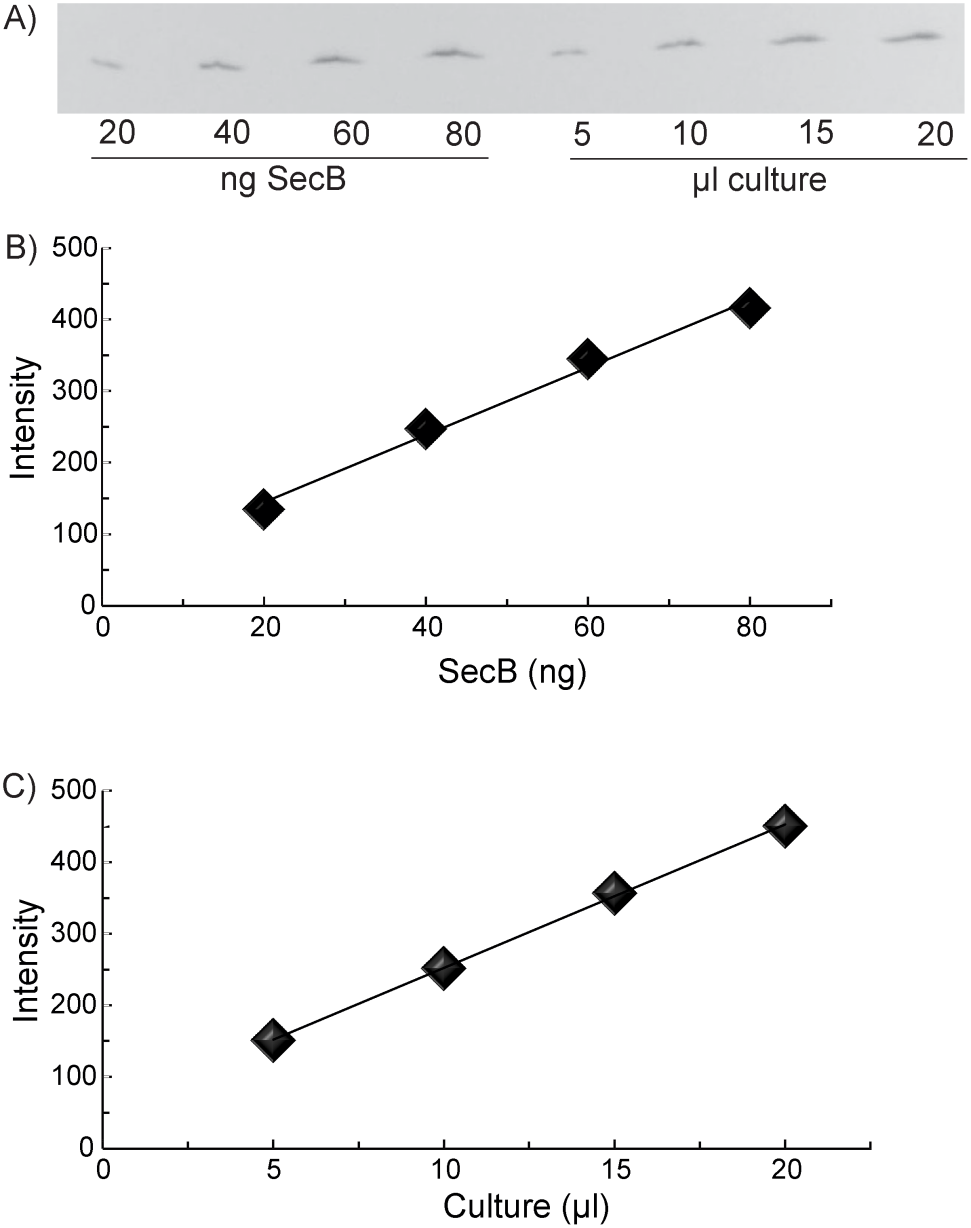
Determination of the cellular concentration of SecB. The culture was grown in minimal medium. (**A)** Immunoblot of a standard curve (lanes 1–4) created by adjusting purified SecB to contain the same amount of total cellular content as that of the unknown sample by addition of a *secB* null cell culture: lanes 1–4 contain purified SecB loaded at 20, 40, 60, 80 ng mixed with cell culture to correspond to amount in lanes 5–8, lanes 5–8 contain the cell culture to be quantified for content of SecB loaded at 5, 10, 15, 20 μl. (**B)** Quantification of the intensity on the immunoblot for the standard, lanes 1–4 in **(A)**. (**C)** Quantification of the intensity for the unknown, lanes 5–8 in **(A)**.

The concentration of intracellular SecB was determined for growth in two media, rich medium (Luria-Bertani broth) and minimal medium (M9) supplemented with 0.4% glycerol [38]. Three independent cultures in each medium were grown at 35°C and subjected to immunoblotting. The amount of SecB in each culture was determined from the ratio of the slope of the standard curve to the slope of the SecB samples to be quantified. The slope of the standard curve (m_std_) is the increment of intensity of signal divided by the change in the mass of SecB: Δ intensity/Δmass (ng). The slope of the SecB sample curve (m_sample_) is the change in the intensity of signal divided by the corresponding change in the volume of sample loaded: Δintensity/Δvolume (μl). Thus, the ratio of the slopes directly gives the concentration of SecB in a given volume of the sample: (m_sample_/m_std_) = ng μl^-1^. For the example shown in Fig 3, the concentration of SecB in the culture, which was grown in minimal medium, was 4.3 ng μl^-1^.

In many publications the amount of protein of interest is expressed per mg of total protein. The total amount of protein per cell has been shown to be constant at approximately 0.41 pg per cell, independent of osmolality of the growth medium and the growth rate [39]. Thus, one can calculate the number of copies per cell. To calculate concentration one needs to know the volume of water per cell. In contrast to protein mass the volume of cellular water decreases as the osmolality of the growth medium increases [40–42]. The osmolalities of the growth media used in this study, given in Table 2, were determined by dew-point temperature depression (see Materials and methods). Knowing the osmolality, we used data presented in Table 1 of Cayley et al. [39] to estimate the total water volume. Total water includes the periplasm as well as the cytoplasm. Changes in osmolality result in changes in the distribution of water between the two cellular compartments [40]. Based on the published data in Fig 2 of Cayley et al. [40], we estimated the fraction of total water that is in the cytoplasm in cells grown at 0.29 Osm as 0.85 and in cells grown at 0.42 Osm as 0.82. Calculations of the molar concentration of cellular SecB used the values in Table 2.

As an example we give the calculations for the experiment shown in Fig 3 in which cells were grown in minimal medium.

Parameters:

OD, the optical density given in absorbance units ml^-1^ at 560 nm
OD_s_, OD_560_ of sample: 15 cells ml^-1^ OD^-1^, number of *E*.*coli* cells at an optical density of 1 at 560 nm, 1.5 × 10^9^ cells ml^-1^ OD^-1^
V_cyto_, volume of cytoplasmic water in an *E*.*coli* cell: 1.3 × 10^−15^ L
C_SecB_, concentration of SecB in the sample: 4.3 × 10^−3^ g L^-1^
V_ts_, total sample volume: 0.85 × 10^−4^ L
Molar mass of SecB tetramer: 69 × 10^3^ g mol^-1^

Calculations:

Number of cells in the sample: *(cells ml*^*-1*^*OD*^*-1*^*) (OD*_*s*_*) (V*_*ts*_*)* = (1.5 × 10^9^ cells ml^-1^ OD^-1^) (15 OD) (0.85 × 10^−4^ L) = 1.9 × 10^9^ cells
Total mass of SecB in the sample: *C*_*SecB*_ *(V*_*ts*_*)* = (4.3 × 10^-3^g L^-1^) (0.85 × 10^−4^ L) = 3.7 × 10^−7^ g
Mass of SecB in one *E*.*coli* cell: (*total mass) (number of E*. *coli cells)*^*-1*^ = (3.7 × 10^−7^ g) (1.9 × 10^9^ cells)^-1^ = 2 × 10^-16^g cell^-1^

Therefore:

Molarity of SecB in the cell: *(mass of SecB g cell*^*-1*^*) [(molar mass of SecB g mol*^*-1*^*) (V*_*cell*_ *L cell*^*-1*^*)]*^*-1*^ = (2 × 10^−16^ g cell^-1^) [(69 × 10^3^ g mol^-1^) (1.3 × 10^−15^ L cell^-1^)]^-1^ = 2.2 μM

Determinations were done for three independent cultures in each of the media. The average concentrations of SecB in the two growth media differed by less than two-fold: 2.5 ± 0.6 μM for minimal medium and 1.6 ± 0.2 μM for rich medium.

## Conclusion

If used properly quantitative Western blotting is a valid technique that is readily accessible to any laboratory. Modern techniques such as proteomic analyses generate large bodies of data which allow investigators in systems biology to develop quantitative models for biological systems. These methods require specialized instrumentation and expertise in downstream bioinformatics analyses and, thus, are currently accessible only to laboratories that are dedicated to proteomics analyses.

In order to achieve quantitative results by immunoblotting of a protein of interest the samples for the standard and those for the unknown must experience the same conditions. Proteins used to generate a standard curve must be mixed with the same quantity of cellular lysate that is present in the experimental samples. This finding has been previously mentioned in the methods of at least two papers in the literature [43, 44]. We emphasize it here in hopes of saving other research groups the necessity of discovering it independently.

We determined the intracellular concentration of SecB expressed from the chromosome in cells growing slowly in minimal medium and in cells growing rapidly in rich medium. We observed a small difference, 2.5 ±0.6 μM SecB tetramer for slow growth and 1.6 ±0.2 μM for rapid growth. Considering the error in the measurements, this is not likely to be significant. These results are in agreement with the conclusion previously published by Seoh and Tai [27] that whereas the levels of other proteins of the Sec system, SecA, SecY and SecE were dependent on the growth rate, the level of SecB was not. However, under a growth condition (minimal medium with glycerol) similar to that of Seoh and Tai [27], we observed a six-fold higher concentration of SecB than that which they reported, 2.5 μM vs 0.4 μM [27]. It is likely that Seoh and Tai prepared the standards for their immunoblots without compensation for the cellular proteins in the sample, which as we show here is essential for quantitative results.

The intracellular concentrations of SecB reported here will allow the extensive body of literature concerning SecB and its interactions with binding partners to be related to export in a living cell.
